# The Book World of Medicine and Science

**Published:** 1900-06-09

**Authors:** 


### The Book World of Medicine and Science.

Heart Disease in Childhood and Youth. By C. W. Chapman, M.D., M.R.C.P., Physician to the National Hospital for Diseases of the Heart, &c. (Pp. 100. Price 3s. 6d. London: The Medical Publishing Company, Limited. 1900.)

As Sir Samuel Wilks remarks in the brief introduction he has written, this little book is one of great interest and value. For many years Dr. Chapman has been much interested in the cardiac diseases of children, and of his skill and insight in their management no one can perhaps better testify than the present writer, whose childhood was passed in a place remote from Wimpole Street, under Dr. Chapman's kindly and observant eye. And it is only right that the observations of one who has spent years of study amongst the incidents of general practice should be recorded and receive the recognition which is due to them. It is peculiarly in complaints such as these which form the subject of this book that the general practitioner acquires an experience that is unrivalled by that of the hospital and consulting physician. Dr. Chapman, instead of attempting an exhaustive study, has elected, we believe wisely, to narrate some typical cases that have been under his care, and to give full and definite details of the particulars of treatment and hygiene practised and enjoined. He incidentally brings into notice the modifications that must be made in our injunctions according to the patient's social status. It is often, unfortunately, a futility to give what must be counsels of perfection; it is a mockery to advise one patient to take rest or another a journey to Nauheim. But for each a little can be done at least, and it is this that Dr. Chapman's wise and discriminating counsels teach us to do.

The Student's Handbook of the Surgery of the Alimentary Canal. By A. Ernest Maylard, M.B., B.S., Lond., Surgeon to the Victoria Infirmary, Glasgow, and Examiner in Surgery to the Victoria University, Manchester. (London: J. and A. Churchill. 1900. Pp. 510; 97 Illustrations. Price 8s. 6d.)

This volume is an abridged and yet supplemented and amended edition of the author's former and now well-known work on the "Surgery of the Alimentary Canal." Mr. Maylard repeats, and this with justice, his belief that much is to be gained by a review of the lesions of nearly the entire length of the alimentary tract in one book, rather than by the discussion of its different component parts in as many treatises. The whole book is thoroughly thoughtful and sound, at the same time being eminently practical and scientific. It is written in its present form chiefly for the use of the student and the busy practitioner. Having briefly described the surgical anatomy and physiology of each portion of the canal at the moment under consideration, the author passes to the pathology of the various lesions that may affect the part, and then deals with the signs and symptoms that they may give rise to, finishing with a clear account of their appropriate treatment. The illustrations in the book are of great merit, consisting to a large extent of reproductions from actual photographs. Due regard has been paid to the newer methods of examination with a view to diagnosis, including the application of skiagraphy to abdominal surgery, especially in the detection of foreign bodies within the stomach and intestines. Peculiarly lucid and accurate are the descriptions of the various operative procedures upon the successive portions of the tract, and the table of the more general operations at present in vogue, given at the commencement of the book, indicates the great advance in the surgery of the digestive system. In gastro-enterostomy the author inclines to the employment of Murphy's anastomosis button, and in his recent cases he avers that he could not have had better results, though this cannot be said to have been the experience of certain other surgeons who have used the same apparatus. The after-treatment of the various operations is shortly but clearly indicated. Perhaps some of the most interesting pages are those on the treatment of appendicitis. Mr. Maylard's graphic account of his simple line of treatment in the early stages of an acute attack should be read with profit by all. He is to be congratulated on the masterly way in which he has handled his subject, and to be thanked for the opportunity that he has given to the student of acquiring a succinct description of this most important branch of surgical knowledge.

The Physical Basis of Memory. By W. Elder, M.D., F.R.C.P. Pp. 24 octavo. Paper, price 6d. (Edinburgh: Oliver and Boyd. 1900.)

In this little pamphlet—a reprint of a lecture delivered before certain associations—Dr. Elder deals pleasantly, if not profoundly, with some of the problems concerning memory. We are more inclined to quarrel with Dr. Elder for the name given to his remarks than with his observations, inasmuch as he only delicately touches the deep and perhaps insoluble mystery of the physical basis of memory. Of what nature is the impression made on a brain cell on the reception of a stimulus? How far can any one cell, when once "impressed," stored with a "memory," be the subject of fresh impressions? What is the cell change that is undergone when "memory" is lost? Can cells involved in any one memory process work at one time or another in different and opposed combinations? These are some of the questions which Dr. Elder, we had hoped, would have helped us to answer. However, Dr. Elder, true Scotsman without doubt, has inclined rather towards metaphysical discussion, and while rejecting the Hamiltonian doctrine of "a faculty of memory," yet speaks of the faculty of attention, and, we fear, fails to recognise the truth of Ribot's discrimination between voluntary and involuntary attention. And we do not think that a hypercritical eye is needed to see that Dr. Elder employs phraseology that may encourage many in the retention of the popular delusion that "Law" is a causative agent, and not merely a precise statement of observed processes and series. However, the pamphlet is interesting and stimulating, and the observations of Dr. Elder on the education of memory are lucid and valuable.

